# *Brettanomyces bruxellensis* population survey reveals a diploid-triploid complex structured according to substrate of isolation and geographical distribution

**DOI:** 10.1038/s41598-018-22580-7

**Published:** 2018-03-07

**Authors:** Marta Avramova, Alice Cibrario, Emilien Peltier, Monika Coton, Emmanuel Coton, Joseph Schacherer, Giuseppe Spano, Vittorio Capozzi, Giuseppe Blaiotta, Franck Salin, Marguerite Dols-Lafargue, Paul Grbin, Chris Curtin, Warren Albertin, Isabelle Masneuf-Pomarede

**Affiliations:** 1 Univ. Bordeaux, ISVV, Unité de recherche Œnologie EA 4577, USC 1366 INRA, Bordeaux, INP 33140 Villenave d’Ornon France; 2 Université de Brest, EA 3882, Laboratoire Universitaire de Biodiversité et Ecologie Microbienne, ESIAB, Technopôle Brest-Iroise, 29280 Plouzané, France; 3 Université de Strasbourg, Centre National de la Recherche Scientifique, Génétique Moléculaire, Génomique, Microbiologie, Unité Mixte de Recherche, 7156 Strasbourg, France; 4 Department of the Science of Agriculture, Food and Environment, University of Foggia, Foggia, Italy; 5 Department of Agricultural Sciences, Division of Vine and Wine Sciences, University of Naples Federico II, Viale Italia, 83100 Avellino, Italy; 6 INRA, UMR Biodiversité Gènes et Ecosystèmes, PlateForme Génomique, 33610 Cestas, France; 7 Bordeaux INP ISVV EA 4577, F-33140 Villenave d’Ornon, France; 8 School of Agriculture, Food and Wine, The University of Adelaide, PMB 1, Glen Osmond, SA 5064 Australia; 9 Department of Food Science and Technology, Oregon State University, 100 Wiegand Hall, Corvallis, Oregon, 97331-6602 USA; 10 ENSCBP, Bordeaux INP, 33600 Pessac, France; 11 Bordeaux Sciences Agro, 33170 Gradignan, France

## Abstract

*Brettanomyces bruxellensis* is a unicellular fungus of increasing industrial and scientific interest over the past 15 years. Previous studies revealed high genotypic diversity amongst *B. bruxellensis* strains as well as strain-dependent phenotypic characteristics. Genomic assemblies revealed that some strains harbour triploid genomes and based upon prior genotyping it was inferred that a triploid population was widely dispersed across Australian wine regions. We performed an intraspecific diversity genotypic survey of 1488 *B. bruxellensis* isolates from 29 countries, 5 continents and 9 different fermentation niches. Using microsatellite analysis in combination with different statistical approaches, we demonstrate that the studied population is structured according to ploidy level, substrate of isolation and geographical origin of the strains, underlying the relative importance of each factor. We found that geographical origin has a different contribution to the population structure according to the substrate of origin, suggesting an anthropic influence on the spatial biodiversity of this microorganism of industrial interest. The observed clustering was correlated to variable stress response, as strains from different groups displayed variation in tolerance to the wine preservative sulfur dioxide (SO_2_). The potential contribution of the triploid state for adaptation to industrial fermentations and dissemination of the species *B. bruxellensis* is discussed.

## Introduction

Grape derived wine is one of the most popular alcoholic beverages and has been produced by humans since ancient times. It is the result of grape juice fermentation by yeasts which consume the fruit sugars and mainly release ethanol and carbon dioxide. Even though microorganisms are an essential part of the winemaking process, they must cope with a very hostile and variable environment, characterised by high initial sugar content and subsequent high ethanol content, low pH, presence of antimicrobial agents and lack of nutrients. Despite these stressful conditions, some opportunistic microorganisms manage to survive and multiply during and after alcoholic fermentation. A striking example is the wine spoilage yeast *Brettanomyces bruxellensis* (teleomorph *Dekkera bruxellensis*) that is typically detected during wine aging but also at lower frequency during the early stages of the winemaking process (grapes and must)^1,2^. When it grows in wine, *B. bruxellensis* produces odorant molecules (namely volatile phenols), which are associated with unpleasant aromas described as barnyard, horse sweat, Band-aid®^3–5^. Therefore, the presence of *B*. *bruxellensis* in wine often provokes rejection by consumers and serious economic losses for winemakers^6^.

The wider industrial relevance of this yeast is highlighted by the fact that it is isolated from various fermented beverages and products. For example, *B. bruxellensis* is an essential contributor to the elaboration of some specialty Belgian and American beers, which are the result of complex spontaneous fermentations performed by various genera of bacteria and yeasts^7,8^. Indeed, *B. bruxellensis* was the first microorganism to be patented for its contribution to English ‘stock’ ales^9^, in 1904. This yeast has also been isolated from other fermented beverages and food like kombucha, kefir, cider, and olives^7,10,11^. Interestingly, *B. bruxellensis* was reported to be a common contaminant in bioethanol production plants^12,13^, and under the right conditions can take the place of the industrial *Saccharomyces cerevisiae* strains and perform molasses fermentation^13^.

The recurrent problem of *B. bruxellensis* in wine and its potential use for beer and bioethanol industrial fermentations has led to high and rising interest in this yeast species. Various studies highlighted great phenotypic diversity of *B. bruxellensis* regarding growth capacity^14–19^, sugar metabolism^20–23^, nitrogen source utilisation^21,24^, volatile phenols production^5,14,18,20,23,25,26^, behaviour in viable but not cultivable state^27^, and response to abiotic factors like temperature^20,28^, pH^20,29^, oxygen availability^30–32^ and sulfur dioxide (SO_2_)^20,23,28,33–35^. This phenotypic variation makes it difficult to predict the spoilage potential of *B. bruxellensis* and is therefore a major concern for winemakers. For example, across several studies the concentration of molecular SO_2_ (mSO_2_) required to stop *B. bruxellensis*’ growth ranged from 0.2 to 1.0 mg.L^−1^ ^36^. This observed variability was at least partly due to the use of different strains. However, only a few studies have attempted to correlate SO_2_ tolerance to a genotypic profile^20,34^. A striking example is a study of 41 *B. bruxellensis* wine isolates from Australia showing that the most common genotype (92% of studied isolates) was correlated with SO_2_ tolerance, thus suggesting that SO_2_ usage patterns may have created a selective pressure on this population^34^.

Despite several studies that have explored genetic diversity of this species using fingerprinting techniques such as Random Amplified Polymorphism DNA (RAPD), Amplified Fragment Length Polymorphism (AFLP), pulsed field electrophoresis (REA-PFGE), and mtDNA restriction analysis^14,17,20,25,26,34,37–40^, our understanding of the *B. bruxellensis* global population structure and the factors that drive it remains limited. Several studies highlight an important intraspecific diversity of *B. bruxellensis*^14,20,38,40^ which makes the prediction of its occurrence and behaviour in industrial fermentations difficult. Further, recent genetic studies on a limited number of strains^24,41,42^ have suggested that polyploidy and hybridisation may play a significant role in microevolution of the species, along with plasticity in chromosomal structure due to “untraditional” centromeres^43^. The role of polyploidy in adaptive changes to suit environment and/or lifestyle has been observed in other organisms^44–47^, notably for *S. cerevisiae* which shares similar fermentation niches to those occupied by *B. bruxellensis*.

To enhance our knowledge of the global *B. bruxellensis* population, here we used a recently developed microsatellite profiling method^42^ to genotype 1488 isolates from various fermentation niches across five continents. Typing based on microsatellite markers is a rapid, reliable and discriminant genotyping approach that has been successfully used to decipher complex population structures^48,49^ and provide insight into the ploidy-state^42^. The performed research work aimed to determine the population structure of a large *B. bruxellensis* collection and test for a link between the identified subpopulations and their adaptive ability, with a focus on tolerance to sulfur dioxide.

## Results

### ***B. bruxellensis*genotyping analysis and population structure**

The *B. bruxellensis* collection used in this study comprised 1488 isolates from 29 countries and 9 different substrates, the majority of strains (87%) originating from wine (Supplementary Table S1). The 1488 isolates were genotyped with 12 primer pairs amplifying microsatellite regions, including four new loci in addition to the eight previously published^42^. Characteristics of the different loci and number of alleles are given in Supplementary Table S2. One locus out of the four additional loci (D1) displayed a high allelic diversity, presenting 18 different alleles. All isolates were shown to be heterozygous for at least one locus. Many isolates were shown to have more than 2 alleles per locus. About half of the isolates had up to 3 alleles per locus (792 isolates) and some had up to 4 and 5 alleles per locus (67 and 1 isolates, respectively). The high number of isolates with up to 3 alleles per locus suggests the existence of triploidy in the studied population. Similar observation was reported previously by Curtin *et al*.^41^ and Borneman *et al*.^24^ who performed *de-novo* sequencing and comparative genomics respectively, highlighting two triploid strains having core diploid genome and additional sets of chromosomes resulting from different triploidisation origins for the two strains. Based on those observations and the occurrence among the isolates of genotypes presenting more than two alleles/locus we extend this hypothesis to the latter.

**Figure 1:**
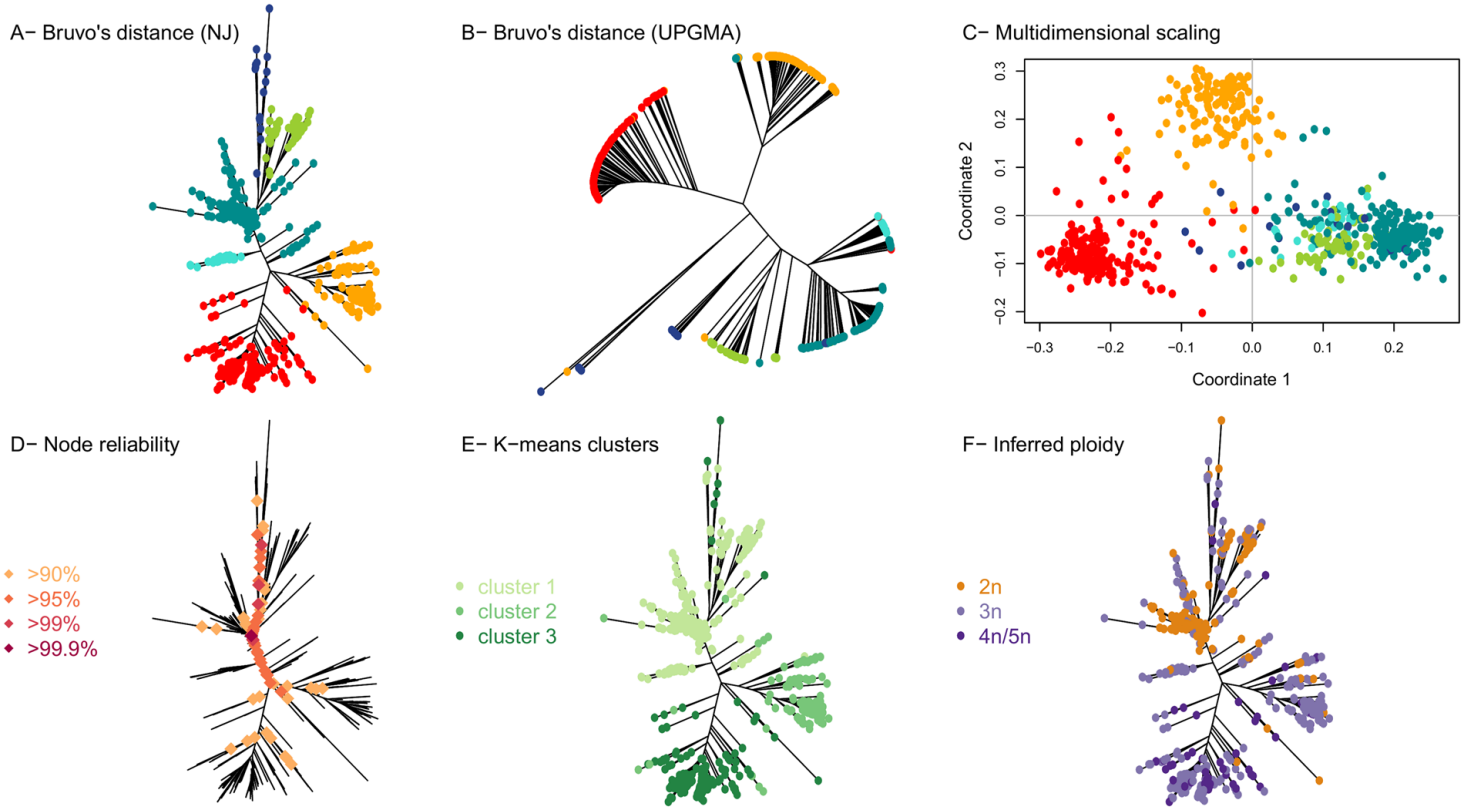
*B. bruxellensis* population clusters identification by combining different tools and parameters. (**A**) Dendrogram using Bruvo’s distance and NJ clustering. The figure was produced using the *poppr* package in R. (**B**) Dendrogram using Bruvo’s distance and UPGMA clustering. The figure was produced using *poppr*. Isolates are shown in the same colours as in A. (**C**) Multidimensional scaling performed with Bruvo’s distance matrix on the same dataset and using the *cmdscale* function on R. For isolates with incomplete genotyping, the missing data was inferred from the closest neighbour using Bruvo’s distance. Isolates are shown with the same colours as in A. (**D**) Node reliability using the partition method^50^. Only the nodes with reliability >90% are shown on the NJ tree. (**E**) Cluster identification using successive K-means. The *find.cluster* function from the *adegenet* package in R was applied, using within-groups sum of squares (WSS) statistics and the default criterion *diffNgroup*. This tool identifies an optimal number of 3 clusters, represented on the NJ tree using different arbitrary colours. (**F**) Inferred ploidy. The maximum number of alleles per locus was computed. Isolates with up to 2 alleles/locus were considered as diploid (2n). Isolates with up to 3 alleles/locus were considered as triploid (3n), and the number of loci showing up to 3 alleles was recorded (1–2 loci, or more than 2 loci showing up to three alleles). Finally, isolates with up to 4 or 5 alleles/locus were noted as 4n/5n. The inferred ploidy is represented on the NJ tree.

The raw data obtained by the microsatellite analysis corresponds to the alleles (*i.e*. the size of the amplified microsatellite sequences) per locus and per strain (Supplementary Table S3). This data was further used for the construction of a dendrogram reflecting the genetic proximity between strains (Fig. 1A). The method was based on Bruvo’s distance and Neighbour Joining (NJ) and was chosen for being reliable and suitable for populations with mixed ploidy levels. The population clusters in 3 main genetic groups (Fig. 1A). Additional methods, including complementary tests and Bayesian approaches were applied to verify the reliability of the clustering obtained by NJ (Fig. 1). The NJ tree showed three main branches that were almost perfectly conserved with UPGMA method (Fig. 1A and B). Then, a multidimensional scaling was performed with Bruvo’s distance matrix on the same dataset and using the *cmdscale* function on R (Fig. 1C). The multidimensional scaling analysis showed that the three main groups were almost identical to the clusters previously defined. Furthermore, the partition method^50^ was applied on the same dataset. This algorithm identifies monophyletic clusters for which the individuals are more closely related than randomly selected individuals. The reliability of the node is then computed and nodes with reliability higher than 90% are considered (Fig. 1D). The partition method also confirmed the three main clusters obtained with NJ as reliable. Finally, clusters were identified using successive K-means (adegenet package, function ‘*find.clusters’*). This function implements the clustering procedure used in Discriminant Analysis of Principal Components (DAPC)^51^, where successive K-means are run with an increasing number of clusters (k), associated with a statistical measure of goodness of fit. This approach identified 3 clusters, once again very similar to those obtained by NJ (Fig. 1E). Overall, the five approaches taken together confirmed the reliability of the three main clusters observed in the studied *B. bruxellensis* population.

**Figure 2:**
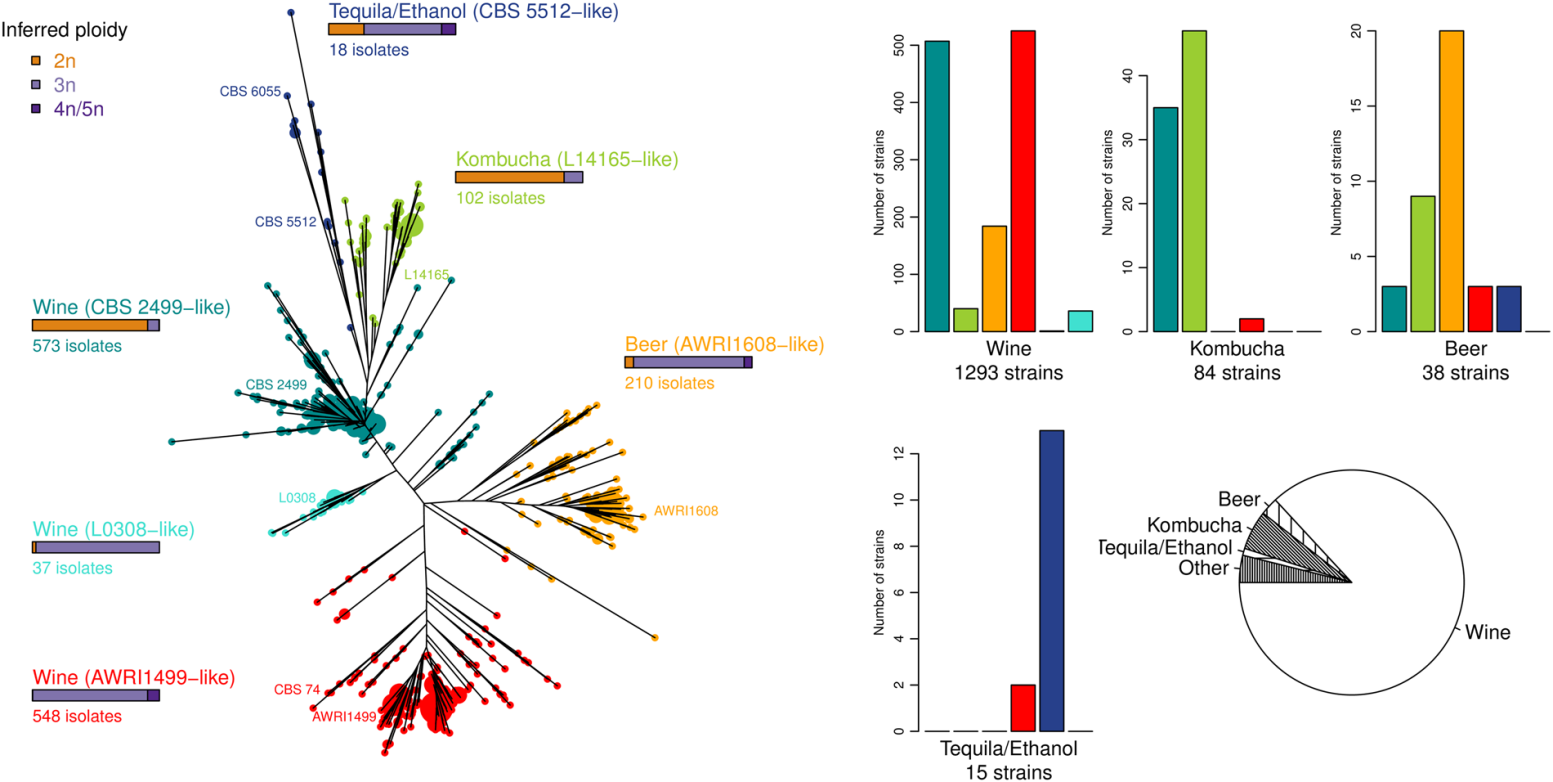
Dendrogram of 1488 isolates of *B. bruxellensis* using 12 microsatellite markers. The dendrogram was drawn *via* the *poppr* package, using Bruvo’s distance and NJ clustering. Five clusters were considered and are represented by different colours. Isolates displaying identical genotypes are represented by a unique tip whose size is proportional to the number of isolates. Inferred ploidy was made as described in Fig. 1F. The histograms represent the distribution of isolates depending on the substrate and the five considered clusters. The pie chart illustrates the proportion of the strains originating from different types of sources.

Since *B. bruxellensis* is known to exhibit different ploidy levels^24,41^, we inferred putative ploidy level based on the microsatellite genotyping. Isolates with up to 2 alleles per locus were considered diploid and noted 2n (Fig. 1F). Isolates with up to 3 alleles/locus were considered triploid (3n). Finally, isolates with up to 4–5 alleles/locus were noted as 4n/5n. The ploidy level coincided clearly with the three main branches of the dendrogram, the red and orange groups being mostly triploid and the blue-green mostly diploid. Within this last cluster, two triploid sub-groups based on the substrate origin and ploidy level of the strains were defined, marked with blue and cyan colours. Finally, the combination of different methods and factors defined of 3 main groups, the ‘diploid’ one being further divided into 3 subgroups (Table 1 and Fig. 2).

**Table 1:** Clusters considered as a result of the microsatellite analysis and cluster validation with five different clustering methods.

Group name	Number of isolates	Number of genotypes	Putative ploidy (for most of the isolates in the group)	Substrate
AWRI1499-like	548	197	Triploid	Mostly from wine
AWRI1608-like	210	127	Triploid	Beer and Wine
CBS 2499-like	573	208	Diploid	Wine
L0308-like	37	26	Triploid	Wine
CBS 5512-like	18	16	Triploid	Bioethanol and tequila
L14165-like	108	58	Diploid	Kombucha

To assess the relative importance of geographical localisation, substrate origin and ploidy level on *B. bruxellensis*’ population structure, an analysis of molecular variance (AMOVA) was performed. The three factors were shown to be significant (p-value < 0.0001). Ploidy level explained 46.9% of the variance, whereas the geographical origin and substrate factors explained only small proportions of the total variation (around 5% for each) (Table 2). However, when considering non-wine isolates, the geographical origin explains 54.8% of the total variance, suggesting that wine genotypes are highly disseminated across the regions studied in comparison with other substrates. The correlation between genetic and geographic distance matrix (MANTEL test) was also significant (p-value = 0.0009), confirming that the genetic variation of the total population is significantly related to geographical localisation. The MANTEL test, performed only on the wine strains (p-value = 0.0040), also confirmed the results obtained with AMOVA, suggesting a different population structure amongst wine strains compared to those from the other niches.

**Table 2:** Impact of geographical localisation, substrate origin and ploidy on the population variance (AMOVA test).

Factor	%Variance	p-value
Country	4.89	<0.0001
Country (wine isolates)	3.7	<0.0001
Country (non-wine isolates)	54.8	<0.0001
Substrate	5.93	<0.0001
Ploidy	46.9	<0.0001

### Core genotype analysis

#### Core diploid data subset

Most classical population genetic analyses cannot be performed using our initial microsatellite dataset since *B. bruxellensis* population include diploid and polyploid isolates, and most traditional analyses are not available for mixed ploidy levels. To overcome such difficulties, we excluded the alleles identified as specific to the isolates showing more than 3 alleles for at least one locus. Among the 124 alleles included in the initial dataset, 70 were found to be significantly associated with the triploid isolates (χ² test, p-value < 0.01), and were excluded to create a new dataset comprising alleles representative of the core genotype (*i.e*. the genotype common to all groups). This approach is justified as previous comparative genomics studies showed that *B. bruxellensis* isolates shared a core diploid genome^24^.

The obtained core genotype dataset showed up to 2 alleles per locus for most individuals (1350 out of 1488) and only 138 remaining individuals had loci with 3 or 4 alleles. This indicates that the removal of specific triploid alleles allowed us to have access to the core diploid genome common to all *B. bruxellensis* isolates. Loci with more than 2 alleles were considered as missing data and only concerned 138 individuals, of which 130 only had one locus with 3 alleles.

#### Ancestral populations and inference of population structure

**Figure 3:**
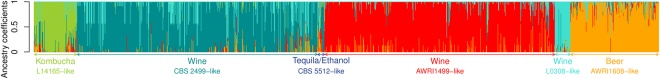
Ancestral populations of 1488 *B. bruxellensis* strains STRUCTURE plots for K = 5 (the number of ancestral population with lowest entropy, see Supplementary Fig. S1). Each bar represents an isolate and the colour of the bar represents the estimated ancestry proportion of each of the K clusters. The same colour code is kept as in Figs 1 and 2.

LEA package and the *snmf* function in R were used to infer population structure for the ‘core diploid’ dataset. The number of ancestral populations tested ranged from K = 1 to K = 15 (100 repetitions), and entropy criterion was computed to choose the number of ancestral populations explaining the genotypic data in the best way (Supplementary Fig. S1). Entropy was minimal for K = 5 ancestral populations (K = 3, 4, 5, 6 shown on Supplementary Fig. S2). Such Bayesian analysis shows that these 5 ancestral populations are congruent with previous analyses that considered the complete dataset (Fig. 3): the AWRI1499-like (wine, red) and AWRI1608-like (beer, orange) groups were associated with only one ancestral population. Likewise, most of the blue-green subgroups (wine CBS 2499-like, wine L0308-like, kombucha L14165-like) previously defined were associated with only one ancestral population. Finally, only the tequila/ethanol group (CBS 5512-like) seemed to be associated with more than one ancestry. Altogether, the population structure analysis on the core diploid genotype confirmed the previous clustering and suggested the existence of only one ancestral population for each current population.

#### Population differentiation analysis

**Figure 4:**
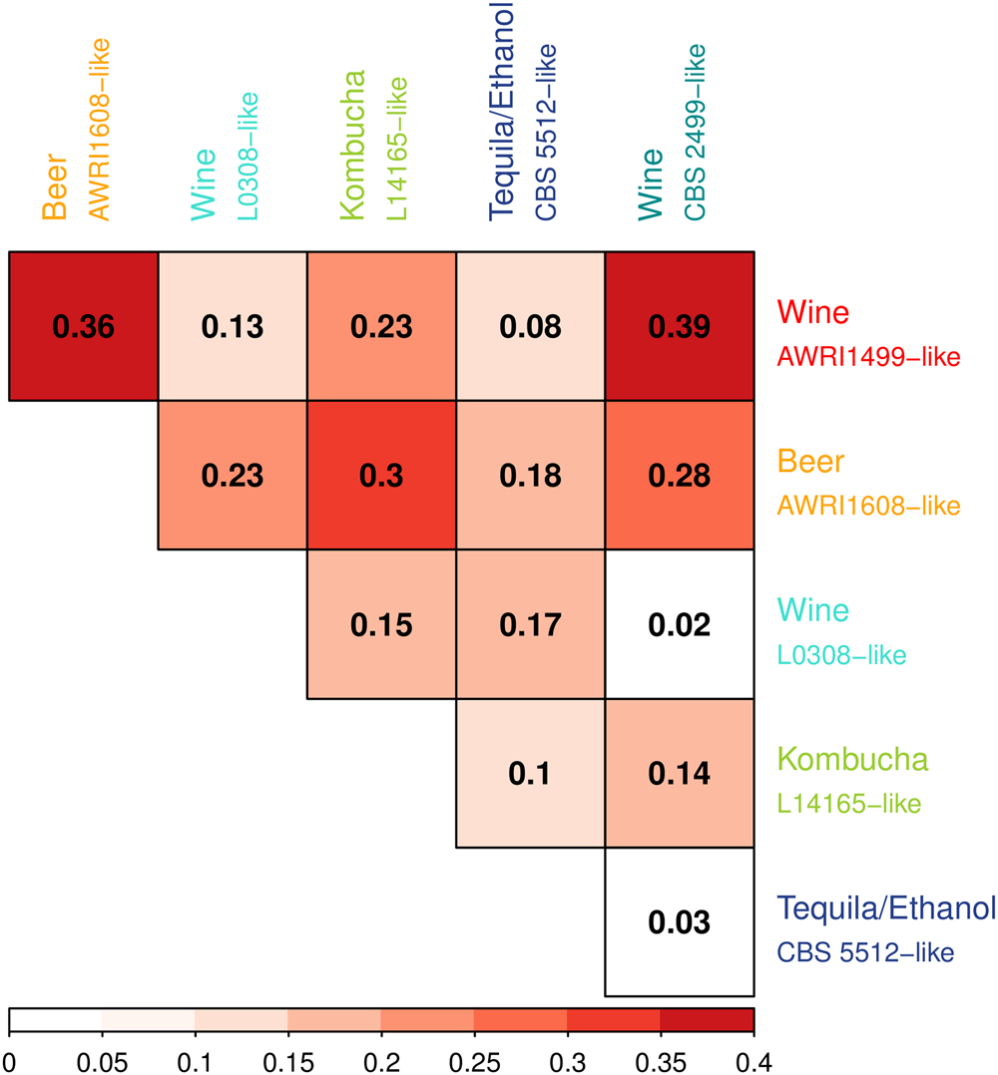
Population differentiation represented by fixation index (*F*_ST_) of *B. bruxellensis* genetic groups between each other. The range of *F*_ST_ is from 0 to 1, 1 meaning that the two populations do not share any genetic diversity.

A population differentiation analysis was performed by calculating the fixation index (*F*_ST_) on the core diploid genotype dataset (Fig. 4). The wine AWRI1499-like population is highly differentiated from beer AWRI1608-like and wine CBS 2499-like groups (with *F*_ST_ 0.36 and 0.39 respectively). This confirms the grouping obtained by the previous analyses. In addition, the pairwise *F*_ST_ values showed high differentiation between beer AWRI1608-like and wine CBS 2499-like populations (*F*_ST_ 0.28). The L14165-like kombucha population seems to be mostly differentiated from the 1608-like beer population and is closer to CBS 5512-like tequila/ethanol group. Finally, it is interesting to point out that the CBS 5512-like group is not highly differentiated from all other groups, which is congruent with the fact that population structure analysis inferred multiple ancestries populations for that group.

### Sulfite tolerance

Sulfur dioxide tolerance was assayed for a subset of *B. bruxellensis* (a total of 39 strains). The chosen strains were selected according to their various geographical origins, substrates and different genetic groups. Some isolates showing identical microsatellite genotypes were included to evaluate possible sulfur tolerance variation between strains with undifferentiated genotypic patterns (13-EN11C11 = L0417 = L0424; UWOPS 92–244.4 = UWOPS 92–262.3; L0469 = L14186). Each strain was grown in medium with increasing SO_2_ concentration (ranging from 0 to 0.6 mg.L^−1^ molecular SO_2_) in biological triplicates, so that more than 480 fermentations were monitored.

**Figure 5:**
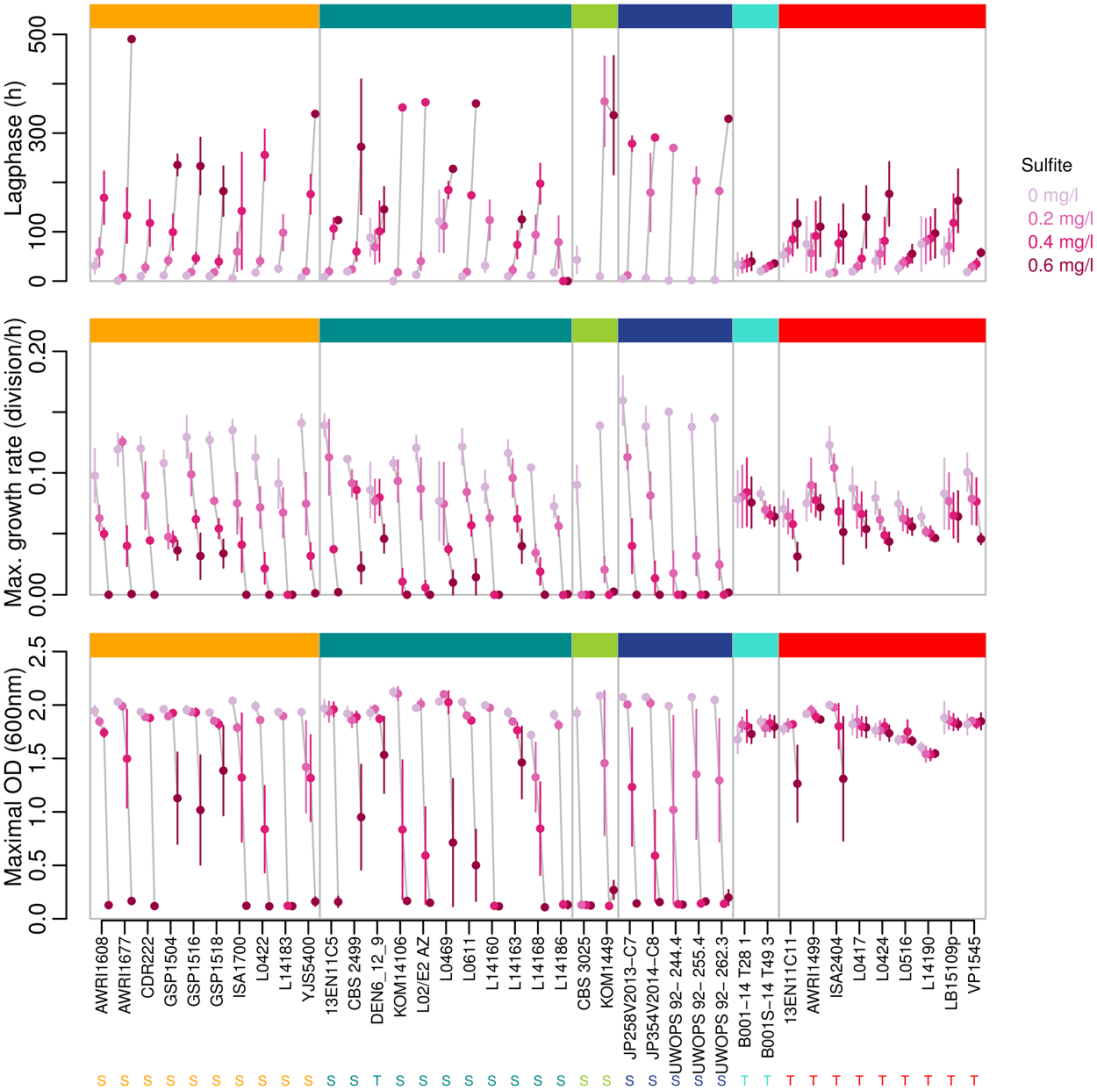
Growth parameters of *B. bruxellensis* strains at different concentrations of SO_2_. 39 strains belonging to the 6 genetic groups defined previously were tested in small scale fermentations and growth (OD_600_) was measured in media containing different concentrations of sulfur dioxide (0, 0.2, 0.4, and 0.6 mg.L^−1^ mSO_2_) and in biological triplicates. Three parameters were considered: lag phase (h): end of lag phase considered when OD above initial OD*5%; maximal growth rate (r) = number of cellular divisions per hour; maximal OD; S and T stand for sensitive and tolerant (Kruskal-Wallis test, α = 5%). Genetic groups are represented in the same colours as on Fig. 2.

Three growth parameters (lag phase, maximum growth rate, maximal OD) in the presence of four different concentrations of mSO_2_ were followed until stationary phase was reached or for a maximum of 300 h when growth was slow or absent. The isolates presented different behaviour according to mSO_2_ concentration (Fig. 5). Based on the growth parameters of the strains when exposed to increased concentrations of mSO_2_, two main groups were identified: (1) sensitive strains (S) characterised by an altered growth with (i) a significant lag phase prolongation, (ii) a significant decrease in maximum growth rate, and/or (iii) significant decrease in maximum OD_600_ (e.g. the sensitive strain L0422 had a lag phase of 17.2 h, 40.7 h, 255.8 h and growth absence, growth rate values were 0.11, 0.07, 0.02 divisions/h and growth absence for and OD_600_ 2, 1.9, 0.8 and no growth at 0, 0.2, 0.4 and 0.6 mg.L^−1^ mSO_2_ respectively); (2) tolerant strains (T) that showed unmodified growth rate and maximum OD_600_ but sometimes a significant prolongation of lag phase was observed (e.g. the tolerant strain AWRI1499 had a maximal growth rate of 0.07, 0.09, 0.08 and 0.07 divisions/h, OD_600_ 1.9, 2.0, 1.9 and 1.9, lag phase of 75, 56.5, 91.5 and 110.3 h at 0, 0.2, 0.4 and 0.6 mg.L^−1^ mSO_2_ respectively for the same strain) (mean values of those parameters for each strain are shown in Supplementary Table S4). A clear relation between genetic group and SO_2_ tolerance was highlighted (Fig. 5). The isolates from groups AWRI1608-like, CBS 5512-like, CBS 2499-like and L14165-like were mostly identified as sensitive (S), whereas the triploid AWRI1499-like and triploid L0308-like groups were mostly classified as tolerant (T). Furthermore, the isolates with an identical microsatellite profile presented similar behaviour in means of growth parameters in the different conditions studied here (Fig. 5 and Supplementary Table S4).

## Discussion

The yeast *B. bruxellensis* has gained importance for its impact not only in wine industry, but also in beer- and bioethanol-associated fermentation processes. Subsequently, many independent studies were held and results were obtained on different *B. bruxellensis* collections but without leading to a holistic picture of the *B. bruxellensis* species. In this study, a large collection of *B. bruxellensis* strains (1488 isolates) from various substrates (9, the majority of strains (87%) being isolated from wine) and geographic origins (5 continents) was genotyped. The use of a reliable and robust method (microsatellite analysis) determined a general picture of the species’ genetic diversity and population structure. The analysis of the complete genotype dataset highlighted 3 main genetic clusters in the *B. bruxellensis* population represented by the AWRI1499-like group, AWRI1608-like and CBS 2499-like group correlating with ploidy level and substrate of isolation. Three sub-clusters were also defined for their ploidy level and substrate of isolation, namely tequila/ethanol CBS 5512-like group, wine L0308-like, and kombucha L14165-like group. Our results are consistent with comparative genomics analysis showing that the AWRI1499, AWRI1608 and AWRI1613 (genetically close to the strain CBS 2499) strains are genetically distant and that the AWRI1499 and AWRI1608 strains are triploid while AWRI1613 is diploid^24^.

Heterozygosity for at least one out of the 12 microsatellite loci was shown for all *B. bruxellensis* isolates. This observation supports the assumption that a simple haploid organisation of the genome is excluded, which is congruent with previous results based on the Southern analysis of single gene probes of 30 *B. bruxellensis* strains from different geographical origins^52^. In comparison, using microsatellite analysis, Legras *et al*. (2007) reported 102 out of 410 *S. cerevisiae* isolates (about 25%) and 75% of *Saccharomyces uvarum* strains (among 108 isolates from various geographical and substrates origins) to be homozygous^53^. In general, highly homozygous strains are associated with sporulation and selfing phenomena^54^. So, this could suggest that in the case of *B. bruxellensis* these mechanisms are non-existent or very rare amongst isolates from industrial fermentation environments. Indeed, there is only one study to our knowledge^55^, which reports spore formation for *B. bruxellensis* (and therefore its teleomorph form *Dekkera bruxellensis*). In the scenario of rare or non-existent sexual reproduction, a large proportion of heterozygous strains would promote higher phenotypic diversity and therefore colonisation of new niches and adaptation to new environments^56^.

Our results confirm on a large scale the assumption that the *B. bruxellensis* population is composed of strains with different ploidy level^24,41,42,52^, as 57.8% of the isolates were shown to have more than 2 alleles for at least one locus. Moreover, polyploid strains were associated with various fermentation niches and geographical regions. A strong correlation between genetic clustering and ploidy level was highlighted, with some clusters predicted to be diploid (CBS 2499-like) while others were composed of mainly triploid isolates (e.g. AWRI1499- and AWRI1608-like). The latter two clusters derive from distinct ancestral populations and thus, presumably from different triploidisation events. The polyploid state typically has a high fitness cost on the eukaryote cell due to the difficulty to maintain imbalanced number of chromosomes during cell division as well as other effects caused by nucleus and cell enlargement^45^. Thus, it is presumed that a stable polyploid or aneuploid state is maintained when it confers advantage for the survival of the cell in particular conditions^47^. Indeed, aneuploidy and polyploidy contribute to genome plasticity and have been shown to confer selective and fitness advantages to fungi in extreme conditions, such as the presence of high concentrations of drugs, high osmotic pressure, low temperature, and others (see^44,47,57^ for review). Similar observations have been made in clinical microbiology, for example, 70% of 132 completely sequenced *S. cerevisiae* clinical isolates with different geographic origins were shown to be poly- or aneuploid^58^. It has been suggested that the aneuploid state contributes to the transition from commercial (industrial fermentations) to clinical (human pathogen lifestyle) environments. Aneuploidy was also reported for another human pathogen – *C. albicans*, for which an aneuploidy of an isochromosome [i(5 L)] is shown to confer resistance to fluconazole^59^. In the industry, stable autotetraploid *S. cerevisiae* strains have been described among isolates from a bakery environment and it was suggested that their prevalence in sour dough fermentation could be the result of human selection for tolerance to high osmotic pressure and high metabolic flux – highly favourable characteristics for baking^60^. In the case of *B. bruxellensis*, however, polyploidy seems to be not only due to a “simple” duplication of chromosomes and/or regions of chromosomes but is the result of independent hybridisation events with closely or distantly related unknown species^24^, which result in allotriploid strains. Efficient hybrid species are not rare in human related fermentations^44,61,62^ and often the hybridisation with a genetically close species is believed to confer tolerance to specific stress factor in a given environment. This is the case of *S. pastorianus*, used for lager beer fermentations characterised with low temperatures. This yeast has recently been shown to be a hybrid between *S. cerevisiae* and *S. eubayanus –* a cryotolerant species isolated from forests in Patagonia^63^, Tibet^64^ and recently from New Zealand^65^. Thus, presumably sterile hybrids were naturally generated and they multiplied clonally, accumulating mutations which enhanced the adaptability of the new “species”^63^. Hybrids are also a widespread state among wine yeast, where natural or laboratory obtained combinations between two species could have interesting technological properties^62,66–69^. Other form of genome dynamics was also highlighted for the diploid CBS 2499 strain possessing specific centromeric loci configuration that enables genome rearrangements and ploidy shifts^43^. Based on the body of knowledge concerning other polyploid micro- and macro-organisms and the prevalence of polyploid strains highlighted in this study, we assume that *B. bruxellensis* has adapted to environmental stress factors by the means of genome plasticity, namely polyploidy.

Our study showed that at least one group, the AWRI1499-like triploid wine group, is composed of wine isolates that are highly tolerant to SO_2_ and that are clearly divergent from other *B. bruxellensis* clusters (*F*_ST_ higher than 0.35 when compared with AWRI1608-like and CBS 2499-like groups). Nevertheless, for some wine samples, isolates from both AWRI1499-like triploid group and the CBS 2499-like diploid group were identified. Coexistence of diploid and polyploid (auto- and allopolyploid) “microspecies” has often been reported for plants, in which the polyploids are widely distributed as opposed to the diploids that have a more restricted distribution^70^. Babcock and Stebbins were the first to name this coexistence of populations a diploid-polyploid complex^71^ for a *Crepis* species defined as a group of interrelated and interbreeding species that also have different levels of ploidy. These authors claimed that such polyploid complex can arise when there are at least two genetically isolated diploid populations and auto- and allopolyploid derivatives that coexist and interbreed. In the case of *B. bruxellensis*, the sexual cycle of this yeast is not yet elucidated and interbreeding remains to be evidenced. However, we propose that *B. bruxellensis* could be described as a diploid-triploid complex, in which sub-populations with different ploidy levels coexist.

To obtain a deeper understanding of the factors shaping *B. bruxellensis* population structure, we explored the impact of geographical localisation and industrial fermentation environment of origin on the total genetic variance of the studied population. Contribution of the “geographic origin” factor to the population structure was shown to be significant yet only explained a relatively small proportion of variation. However, the variance proportion explained by this factor is much higher when considering non-wine isolates, suggesting that wine strains are highly dispersed worldwide. This dispersal could easily reflect exchange of material and human transport associated with winemaking, followed by adaptation to local winemaking practices^38^. Exchange of material also happens between different industries, which would facilitate local transfer of microorganisms between beverages. For example, some beers are aged in oak barrels previously used for winemaking^72^. Also, in the past, beer fermentation is thought to have been initiated by the addition of a small amount of wine^73^. Such exchanges could be a possible explanation for the low (but significant) contribution of the “substrate of isolation” factor to the total genetic variance in the studied population (5.93%, p-value < 0.0001). Substrate of isolation and geographic origin contributed to a similar extent to the total genetic variance of the population. However, this percentage remained low (5%) compared to *S. cerevisiae* for which geographic origin was shown to contribute to 28% of the genetic variance^53^, and *Candida albicans* for which 39% were reported^74^. For *S. cerevisiae*, a significant contribution of geographic origin to the genetic variance is often perceived as a sign of local domestication^53,75^. Like *S. cerevisiae, B. bruxellensis* is isolated from human-conducted fermentations including beer and wine. However, until now there are no *B. bruxellensis* isolates from “natural” non-human related habitats contrary to the case of *S. cerevisiae*^76–78^. A recent comparative study of strains with different industrial origins and their growth capacities in various type of media (wine, beer, and soft drink) suggests adaptation of *B*. *bruxellensis* strains to different fermented beverages^23^. In our study, a low but significant contribution of substrate of isolation to the total genetic variance of the species was highlighted (5.93%, p-value < 0.0001), which is an indicator for the adaptation of certain sub-groups to different human-related niches (e.g. winemaking conditions, kombucha fermentation, and others). This structuration is further accompanied by a specific genetic configuration, some groups being mostly diploid and others polyploid.

The hypothesis that the triploid state of *B. bruxellensis* is maintained for some genetic groups because of its contribution to adaptation to a certain type of environment or stress factors is strongly supported by the sulfite tolerance assay performed in our study. This indicated that strains representative of the globally dispersed wine triploid AWRI1499-like group are highly tolerant to SO_2_. Sulfur dioxide is the most common antimicrobial agent used in winemaking. However, very tolerant *B. bruxellensis* strains have been reported^36^. Particularly, in Australia 92% of the isolates are genetically close to a strain that has be shown to be triploid by genome sequencing and highly tolerant to SO_2_ (normal growth at more than 0.6 mg.L^−1^ mSO_2_)^34^. Here, we show that isolates from this genetic group are highly represented worldwide, namely in France, Italy, Portugal, Southern Argentina and Chile. Furthermore, we confirmed on a larger scale (39 strains from different geographical and fermentation niches) that even high SO_2_ doses could not guarantee the absence of growth of these strains and therefore their potential to spoil wine. In this context, it is worth noting that isolates from substrates other than wine, were all sensitive to SO_2_ which suggests a direct link between SO_2_ exposure in wine and tolerance to this compound. Survival in the presence of SO_2_ has been broadly studied in *S. cerevisiae* but is still not fully elucidated. Molecular SO_2_ was reported to be the major active antiseptic species of SO_2_ in wine by different authors (see review of Divol *et al*., 2012) whereas bisulfites species could also play a role at minor level, in the biocidic effect of PMB^79^. Molecular SO_2_ could enter the cell passively or *via* selective transport^80^. Once inside the cell, molecular SO_2_ at approximate intracellular pH 5.5–6.5, rapidly dissociates into bisulphite and sulphite anions. Then, bisulphite is the dominant and main antimicrobial species of SO_2_ inside the cell that can interact with different enzymes and molecules thus having an impact on the basic metabolic pathways of the cell, such as glycolysis. Strategies to tolerate SO_2_ are also numerous, like its action on the cell: through the production of molecules that bind SO_2_ (acetaldehyde, pyruvate, and others), SO_2_ oxidation and SO_2_ active efflux by sulfite pump (*SSU1*)^80^. Even if in *B. bruxellensis* these mechanisms are not elucidated, SO_2_ tolerance could be linked to different aspects – presence of gene(s) coding for a sulfite transporter or presence of this gene (or genes) in multiple copies and therefore overexpression, differences in the gene regulation leading to more efficient response to SO_2_ toxicity, or morphological and physiological state of the cell that would give it the ability to tolerate this antimicrobial agent (cell membrane structure, growth, *etc*.). The fact that all the highly tolerant *B.bruxellensis* strains are triploid indicates that this genetic configuration could contribute to SO_2_ tolerance. As mentioned in the previous paragraphs, polyploid states are maintained when they confer a selective advantage. In this case, we can hypothesise that the allotriploid AWRI1499-like strains combine genetic and physiological characteristics from the parent genomes that confer to them the ability to survive in the presence of SO_2_.

A possible strategy to cope with the issue of highly tolerant strains would be the increase of SO_2_ concentration added to the must and wine. However, the strong legislation and consumer pressure to reduce any kind of wine additives makes it undesirable to produce wines with high concentrations of SO_2_ which would be needed for the prevention of AWRI1499-like strains growth. Therefore, the genetic content of *B. bruxellensis* has to be considered when choosing spoilage prevention and treatment methods in the winery in order to obtain optimal effect with minimum intervention. Overall, our results show that polyploid strains are widely disseminated and suggest that *B. bruxellensis* is a diploid-triploid complex whose population structure has been influenced by the use of sulfur dioxide as a preservative in winemaking. Thus, we highlight the importance of *B. bruxellensis* species as a non-conventional model microorganism for the study of polyploidy as an adaptation mechanism to human-related environments.

## Materials and Methods

### Yeast strains

*B. bruxellensis* strains used in this study were collected from different origins: (i) from CRB Oenologie collection (Centre de Ressources Biologiques Oenologie, Institut des Sciences de la Vigne et du Vin, France), (ii) sent from other laboratories, and (iii) isolated from wines for the purpose of this work. Overall, the collection of *B. bruxellensis* used in this study contained 1488 isolates (Supplementary Table S1) which were further analysed by genotyping.

Strain isolation from contaminated wines was performed by spreading 100 µL of wine sample on solid YPD medium containing 10 g.L^−1^ yeast extract (Difco Laboratories, Detroit M1), 10 g.L^−1^ bactopeptone (Difco Laboratories, Detroit M1), 20 g.L^−1^ D-glucose (Sigma-Aldrich) and 20 g.L^−1^ agar (Sigma-Aldrich). This medium was supplemented with antibiotics in order to limit the growth of bacteria (5 g.L^−1^ chloramphenicol Sigma-Aldrich), moulds (7.5 g.L^−1^ biphenyl, Sigma-Aldrich), and yeast of the *Saccharomyces* genus (50 g.L^−1^ cycloheximide, Sigma-Aldrich). The samples were then incubated at 30 °C for 5 to 10 days. Ten colonies were then picked randomly and analysed by PCR using the DB1/DB2 primers^81^ (Eurofins MWG Operon, Les Ulis, France) for species identity confirmation (DNA extraction was performed as described below for the microsatellite analysis). Putative *B. bruxellensis* colonies were streaked and grown on selective YPD medium twice consecutively in order to insure the strain purity. Colonies that gave a positive result by PCR DB1/DB2 were stored at -80 °C in 50% YPD/glycerol medium.

### Genotyping by microsatellite analysis

#### DNA extraction

For DNA extraction, strains were grown on YPD solid medium at 30 °C for 5 to 7 days and fresh colonies were lysed in 30 µL of 20 mM NaOH solution heated at 99 °C for 10 minutes using iCycler thermal cycler (Biorad, Hercules, CA, USA).

#### Microsatellite loci identification and primers design

Twelve pairs of primers were designed on the basis of the *de-novo* genome assembly of the triploid *B. bruxellensis* strain AWRI1499^41^ as previously described by Albertin *et al*.^42^. Four pairs of primers were added to the eight that were previously described in order to improve the discriminative power of the test and to insure its robustness (Supplementary Table S2).

#### Microsatellites amplification

In order to reduce the time and cost of analysis, some of the PCR reactions were multiplexed as shown in the Tm column in Supplementary Table S2. By this procedure the number of PCR reactions per sample was reduced from 12 to 9.

PCR reactions were performed in a final volume of 15 µL containing 1 µL of DNA extract (extraction performed as described above), 0.05 µM of forward primer, 0.5 µM of reverse primer and labelled primer (or 1 µL in the case of duplex PCR reactions), 1×Taq-&GO (MP Biomedicals, Illkirch, France). The forward primers were tailed on their 5′ end with M13 sequence as described by Schuelke *et al*.^82^. Universal M13 primers were labelled with FAM-, HEX-, AT565- (equivalent to PET) or AT550- (equivalent to NED) fluorescent dies (Eurofins MWG Operon, Les Ulis, France). This method allows labelling of several microsatellite marker primers with the same fluorochrome marked primer (M13) instead of marking each of the 12 forward primers and thus reduces significantly the analysis cost.

Touch-down PCR was carried out using an iCycler thermal cycler (Biorad, Hercules, CA, USA). The program consisted of an initial denaturation step of 1 min at 94 °C followed by 10 cycles of 30 s at 94 °C, 30 s at Tm + 10 °C (followed by a 1 °C decrease per cycle until Tm is reached) and 30 s at 72 °C, then 20 cycles of 30 s at 94 °C, 30 s at Tm and 30 s at 72 °C, and a final extension step of 2 min at 72 °C.

Amplicons were first analysed by a microchip electrophoresis system (MultiNA, Shimadzu) and the optimal conditions for PCR amplifications were assessed. Then, the exact sizes of the amplified fragments were determined using the ABI3730 DNA analyser (Applied Biosystems) (a core facility of INRA, UMR Biodiversité Gènes et Ecosystèmes, PlateForme Génomique, 33610 Cestas, France). Prior to the ABI3730 analysis, PCR amplicons were diluted (1800-fold for FAM, 600-fold for HEX, 1200-fold for AT565 and 1800-fold for AT550) and multiplexed in formamide. The LIZ 600 molecular marker (ABI GeneScan 600 LIZ Size Standard, Applied Biosystems) was diluted 100-fold and added to each multiplex. Before loading, diluted amplicons were heated 4 min at 94 °C. Allele size was recorded manually using GeneMarker Demo software V2.2.0 (SoftGenetics).

#### Microsatellite data analysis

To investigate the genetic relationships between strains, the microsatellite dataset was analysed using the Poppr package^83^ in R (3.1.3 version, https://www.r-project.org). A dendrogram was established using Bruvo’s distance^84^ and Neighbour Joining (NJ) clustering^85^. Bruvo’s distance takes into account the mutational process of microsatellite loci and is well adapted to populations with mixed ploidy levels and is therefore suitable for the study of the *B. bruxellensis* strain collection used in this work. Supplementary tests were applied to the same dataset in order to confirm the clusters obtained by Neighbour Joining. First, an UPGMA (Unweighted Pair Group Method with Arithmetic Mean) analysis was compared with NJ. Then, the partition method^50^ was applied in order to confirm the reliability of the nodes obtained by NJ. Also, a multidimensional scaling was performed with Bruvo’s distance matrix on the same dataset and using the *cmdscale* function on R and finally, the function ‘*find.clusters*’ available in the adegenet R package was used to identify clusters by successive K-means^86^. Further, AMOVA (analysis of molecular variance) was used to assess the relative importance of geographical localisation and substrate origin regarding *B. bruxellensis* genetic diversity. To confirm the results obtained by the AMOVA analysis, the link between genetic divergence and geographic distance was further evaluated by MANTEL test.

#### Core genotype analysis

Among the 124 alleles included in the initial dataset, 70 were found to be significantly associated with the triploid isolates (χ² test, p < 0.01) and were excluded to create a new dataset comprising alleles common to all groups and representative of the core genotype (*i.e*. the genotype common to all groups).

For the inference of population structure with this dataset, LEA package was used^87^ in combination with the TESS tool to map the geographical cluster assignments of the ancestral populations as defined by Höhna *et al*.^88^. Further, a differentiation test analysis was performed by calculating the fixation index (*F*_ST_) for the core diploid genotype.

### Sulfite tolerance assessment

The assay was performed in liquid medium containing 6.7 g.L^−1^ of YNB (Difco^TM^ Yeast Nitrogen Base, Beckton, Dickinson and Company), 2.5 g.L^−1^ D-glucose, 2.5 g.L^−1^ D-Fructose, 5% (v/v) ethanol and increasing concentrations of potassium metabisulfite (PMB, K_2_S_2_O_5_)(Thermo Fischer Scientific) in order to obtain 0, 0.2, 0.4 and 0.6 mg.L^−1^ mSO_2_ final concentrations. For the calculation of mSO_2_ it was considered that K_2_S_2_0_5_ corresponds to about 50% of total SO_2_ (therefore a solution of 10 g.L^−1^ K_2_S_2_0_5_ corresponds to approximately 5 g.L^−1^ total SO_2_). In order to deduce the final mSO_2_ concentration, the free SO_2_ concentration was assessed by aspiration/titration method. Then, the mSO_2_ was calculated by using the Henderson-Hasselbalch equation on dissociation constant p*K*1^89^. Final pH was adjusted to 3.5 (corresponding to an average value for pH generally encountered in red winemaking conditions) with phosphoric acid (1 M H_3_PO_4_) and the four media (corresponding to the 4 different concentrations of SO_2_) were filtered separately with 0.22 µm pore filter (Millipore).

Small-scale fermentations were performed in sterile 4 ml spectrophotometer cuvettes containing a sterile magnet stirrer (Dutscher, France). The cells were grown on YPD agar and inoculated into the YNB-based medium without SO_2_. After 96 h of pre-culture (the point at which all strains reached stationary phase), the cells were inoculated at OD_600_ 0.1 in a final volume of 3 ml. The inoculated medium was then covered with 300 µL of sterile silicone oil (Sigma-Aldrich) to avoid oxidation of the medium which could favour the free SO_2_ consumption. Then, the cuvette was capped with a plastic cap (Dutscher) and sealed with parafilm. A sterile needle was added by piercing the cap to allow CO_2_ release. The “nano-fermenters” were then placed in a spectrophotometer cuvettes container box and on a 15 multi-positions magnetic stirrer plate at 25 °C (the final temperature in the “nano-fermenters” was therefore 29 °C due to the stirrer heating). Optical density (OD_600_) was measured every 24 h during at least 300 h to follow cell population growth until stationary phase was reached.

For each growth curve, the following three parameters were calculated: maximal OD was the maximal OD reached at 600 nm, the lag phase (in hours) was the time between inoculation and the beginning of cell growth (5% maximal OD increase), and finally, the maximal growth rate was calculated (maximal number of division per hour based on the OD measurement divided by time). A non-parametric Kruskal-Wallis test was used at α = 5% to identify the means that were significantly different.

### Data availability

The datasets generated and analysed during the current study are available from the corresponding author on reasonable request.

## Electronic supplementary material

Supplementary information

Supplementary Table S1

Supplementary Table S2

Supplementary Table S3

Supplementary Table S4

## References

[1] Renouf V, Lonvaud-Funel A (2007). Development of an enrichment medium to detect Dekkera/Brettanomyces bruxellensis, a spoilage wine yeast, on the surface of grape berries. Microbiol. Res..

[2] Renouf, V. Evidence for differences between B. bruxellensis strains originating from an enological environment. *Int. J. Wine Res*. 95 10.2147/IJWR.S4612 (2009).

[3] Chatonnet P, Dubourdieu D, Boidron JN (1995). The Influence of Brettanomyces/Dekkera sp. Yeasts and Lactic Acid Bacteria on the Ethylphenol Content of Red Wines. Am. J. Enol. Vitic..

[4] Chatonnet P, Dubourdieu D, Boidron JN (1992). Incidence des conditions de fermentation et d’élevage des vins blancs secs en barriques sur leur composition en substances cédées par le bois de chêne. Sci. Aliments.

[5] Heresztyn T (1986). Metabolism of volatile phenolic compounds from hydroxycinnamic acids byBrettanomyces yeast. Arch. Microbiol..

[6] Wedral D, Shewfelt R, Frank J (2010). The challenge of Brettanomyces in wine. LWT - Food Sci. Technol..

[7] Steensels J (2015). Brettanomyces yeasts — From spoilage organisms to valuable contributors to industrial fermentations. Int. J. Food Microbiol..

[8] Bokulich NA, Bamforth CW, Mills DA (2012). Brewhouse-Resident Microbiota Are Responsible for Multi-Stage Fermentation of American Coolship Ale. PLOS ONE.

[9] Claussen NH (1904). On a Method for the Application of Hansen’s Pure Yeast System in the Manufacturing of Well-Conditioned English Stock Beers. J. Inst. Brew..

[10] Schifferdecker AJ, Dashko S, Ishchuk OP, Piškur J (2014). The wine and beer yeast Dekkera bruxellensis. Yeast Chichester Engl..

[11] Coton, M. *et al*. Unraveling microbial ecology of industrial-scale Kombucha fermentations by metabarcoding and culture-based methods. *FEMS Microbiol. Ecol*. **93** (2017). 10.1093/femsec/fix048 28430940

[12] Passoth V, Blomqvist J, Schnürer J (2007). Dekkera bruxellensis and Lactobacillus vini Form a Stable Ethanol-Producing Consortium in a Commercial Alcohol Production Process. Appl. Environ. Microbiol..

[13] Souza RBde (2012). The consequences of Lactobacillus vini and Dekkera bruxellensis as contaminants of the sugarcane-based ethanol fermentation. J. Ind. Microbiol. Biotechnol..

[14] Agnolucci M (2009). Genetic diversity and physiological traits of Brettanomyces bruxellensis strains isolated from Tuscan Sangiovese wines. Int. J. Food Microbiol..

[15] Barbin P, Cheval J-L, Gilis J-F, Strehaiano P, Taillandier P (2008). Diversity in spoilage yeast dekkera/Brettanomyces bruxellensis isolated from French red wine. Assessment during fermentation of synthetic wine medium. J. Inst. Brew..

[16] Fugelsang KC, Zoecklein BW (2003). Population Dynamics and Effects of Brettanomyces bruxellensis Strains on Pinot noir (Vitis vinifera L.) Wines. Am. J. Enol. Vitic..

[17] Oelofse A, Lonvaud-Funel A, du Toit M (2009). Molecular identification of Brettanomyces bruxellensis strains isolated from red wines and volatile phenol production. Food Microbiol..

[18] Romano A, Perello Mc, Revel Gde, Lonvaud-Funel A (2008). Growth and volatile compound production by Brettanomyces/Dekkera bruxellensis in red wine. J. Appl. Microbiol..

[19] Vigentini I (2008). Physiological and oenological traits of different Dekkera/Brettanomyces bruxellensis strains under wine-model conditions. FEMS Yeast Res..

[20] Conterno L, Joseph CML, Arvik TJ, Henick-Kling T, Bisson LF (2006). Genetic and Physiological Characterization of Brettanomyces bruxellensis Strains Isolated from Wines. Am. J. Enol. Vitic..

[21] Crauwels S (2015). Comparative phenomics and targeted use of genomics reveals variation in carbon and nitrogen assimilation among different Brettanomyces bruxellensis strains. Appl. Microbiol. Biotechnol..

[22] Galafassi S (2011). Dekkera/Brettanomyces yeasts for ethanol production from renewable sources under oxygen-limited and low-pH conditions. J. Ind. Microbiol. Biotechnol..

[23] Crauwels, S. *et al*. Fermentation assays reveal differences in sugar and (off-) flavor metabolism across different *Brettanomyces bruxellensis* strains. *FEMS Yeast Res*. **17** (2017). 10.1093/femsyr/fow105 27956491

[24] Borneman, A. R., Zeppel, R., Chambers, P. J. & Curtin, C. D. Insights into the *Dekkera bruxellensis* Genomic Landscape: Comparative Genomics Reveals Variations in Ploidy and Nutrient Utilisation Potential amongst Wine Isolates. *PLoS Genet*. **10** (2014). 10.1371/journal.pgen.1004161 PMC3923673 24550744

[25] Di Toro MR (2015). Intraspecific biodiversity and ‘spoilage potential’ of Brettanomyces bruxellensis in Apulian wines. LWT - Food Sci. Technol.

[26] Martorell P (2006). Molecular typing of the yeast species Dekkera bruxellensis and Pichia guilliermondii recovered from wine related sources. Int. J. Food Microbiol..

[27] Capozzi V (2016). Viable But Not Culturable (VBNC) state of Brettanomyces bruxellensis in wine: New insights on molecular basis of VBNC behaviour using a transcriptomic approach. Food Microbiol..

[28] Barata A (2008). Survival patterns of Dekkera bruxellensis in wines and inhibitory effect of sulphur dioxide. Int. J. Food Microbiol..

[29] Blomqvist J, Eberhard T, Schnürer J, Passoth V (2010). Fermentation characteristics of Dekkera bruxellensis strains. Appl. Microbiol. Biotechnol..

[30] Capusoni, C. *et al*. Effects of oxygen availability on acetic acid tolerance and intracellular pH in *Dekkera bruxellensis*. *Appl. Environ. Microbiol*. AEM.00515–16 10.1128/AEM.00515-16 (2016). 10.1128/AEM.00515-16 PMC4984296 27235432

[31] Du Toit Wj, Pretorius I (2005). s. & Lonvaud-Funel, A. The effect of sulphur dioxide and oxygen on the viability and culturability of a strain of Acetobacter pasteurianus and a strain of Brettanomyces bruxellensis isolated from wine. J. Appl. Microbiol..

[32] Uscanga MGA, Délia M-L, Strehaiano P (2003). Brettanomyces bruxellensis: effect of oxygen on growth and acetic acid production. Appl. Microbiol. Biotechnol..

[33] Agnolucci M (2010). Sulphur dioxide affects culturability and volatile phenol production by Brettanomyces/Dekkera bruxellensis. Int. J. Food Microbiol..

[34] Curtin C, Kennedy E, Henschke PA (2012). Genotype-dependent sulphite tolerance of Australian Dekkera (Brettanomyces) bruxellensis wine isolates. Lett. Appl. Microbiol..

[35] Vigentini I, Joseph CML, Picozzi C, Foschino R, Bisson LF (2013). Assessment of the Brettanomyces bruxellensis metabolome during sulphur dioxide exposure. FEMS Yeast Res..

[36] Curtin C, Varela C, Borneman A (2015). Harnessing improved understanding of Brettanomyces bruxellensis biology to mitigate the risk of wine spoilage. Aust. J. Grape Wine Res..

[37] Campolongo S, Rantsiou K, Giordano M, Gerbi V, Cocolin L (2010). Prevalence and Biodiversity of Brettanomyces bruxellensis in Wine from Northwestern Italy. Am. J. Enol. Vitic..

[38] Curtin CD, Bellon JR, Henschke PA, Godden PW, De BL (2007). Genetic diversity of Dekkera bruxellensis yeasts isolated from Australian wineries. FEMS Yeast Res..

[39] Joseph CML, Gorton LW, Ebeler SE, Bisson LF (2013). Production of Volatile Compounds by Wine Strains of Brettanomyces bruxellensis Grown in the Presence of Different Precursor Substrates. Am. J. Enol. Vitic..

[40] Vigentini I (2012). Intraspecific variations of Dekkera/Brettanomyces bruxellensis genome studied by capillary electrophoresis separation of the intron splice site profiles. Int. J. Food Microbiol..

[41] Curtin CD, Borneman AR, Chambers PJ, Pretorius IS (2012). De-Novo Assembly and Analysis of the Heterozygous Triploid Genome of the Wine Spoilage Yeast Dekkera bruxellensis AWRI1499. PLOS ONE.

[42] Albertin W (2014). Development of microsatellite markers for the rapid and reliable genotyping of Brettanomyces bruxellensis at strain level. Food Microbiol..

[43] Ishchuk OP (2016). Novel Centromeric Loci of the Wine and Beer Yeast Dekkera bruxellensis CEN1 and CEN2. PLOS ONE.

[44] Albertin W, Marullo P (2012). Polyploidy in fungi: evolution after whole-genome duplication. Proc. Biol. Sci..

[45] Comai L (2005). The advantages and disadvantages of being polyploid. Nat. Rev. Genet..

[46] Selmecki AM (2015). Polyploidy can drive rapid adaptation in yeast. Nature.

[47] Wertheimer NB, Stone N, Berman J (2016). Ploidy dynamics and evolvability in fungi. Phil Trans R Soc B.

[48] Vieira MLC, Santini L, Diniz AL, Munhoz C (2016). de F. Microsatellite markers: what they mean and why they are so useful. Genet. Mol. Biol..

[49] Guichoux E (2011). Current trends in microsatellite genotyping. Mol. Ecol. Resour..

[50] Prosperi MCF (2011). A novel methodology for large-scale phylogeny partition. Nat. Commun..

[51] Jombart T, Devillard S, Balloux F (2010). Discriminant analysis of principal components: a new method for the analysis of genetically structured populations. BMC Genet..

[52] Hellborg L, Piškur J (2009). Complex Nature of the Genome in a Wine Spoilage Yeast, Dekkera bruxellensis. Eukaryot. Cell.

[53] Legras J-L, Merdinoglu D, Cornuet J-M, Karst F (2007). Bread, beer and wine: Saccharomyces cerevisiae diversity reflects human history. Mol. Ecol..

[54] Mortimer RK, Romano P, Suzzi G, Polsinelli M (1994). Genome renewal: A new phenomenon revealed from a genetic study of 43 strains of Saccharomyces cerevisiae derived from natural fermentation of grape musts. Yeast.

[55] Walt JPVder, Kerken AEV (1960). The wine yeasts of the Cape. Antonie Van Leeuwenhoek.

[56] Magwene, P. M. Revisiting Mortimer’s Genome Renewal Hypothesis: Heterozygosity, Homothallism, and the Potential for Adaptation in Yeast. In *Ecological Genomics* (eds Landry, C. R. & Aubin-Horth, N.) 37–48 (Springer Netherlands, 2014). 10.1007/978-94-007-7347-9_3. 10.1007/978-94-007-7347-9_3 PMC4096854 24277294

[57] Mulla W, Zhu J, Li R (2014). Yeast: a simple model system to study complex phenomena of aneuploidy. FEMS Microbiol. Rev..

[58] Zhu YO, Sherlock G, Petrov DA (2016). Whole Genome Analysis of 132 Clinical Saccharomyces cerevisiae Strains Reveals Extensive Ploidy Variation. G3 Genes Genomes Genet..

[59] Selmecki A, Forche A, Berman J (2006). Aneuploidy and isochromosome formation in drug-resistant Candida albicans. Science.

[60] Albertin W (2009). Evidence for autotetraploidy associated with reproductive isolation in Saccharomyces cerevisiae: towards a new domesticated species. J. Evol. Biol..

[61] Querol A, Bond U (2009). The complex and dynamic genomes of industrial yeasts. FEMS Microbiol. Lett..

[62] Steensels J (2014). Improving industrial yeast strains: exploiting natural and artificial diversity. FEMS Microbiol. Rev..

[63] Libkind D (2011). Microbe domestication and the identification of the wild genetic stock of lager-brewing yeast. Proc. Natl. Acad. Sci..

[64] Bing J, Han P-J, Liu W-Q, Wang Q-M, Bai F-Y (2014). Evidence for a Far East Asian origin of lager beer yeast. Curr. Biol..

[65] Gayevskiy V, Goddard MR (2016). Saccharomyces eubayanus and Saccharomyces arboricola reside in North Island native New Zealand forests. Environ. Microbiol..

[66] Le Jeune C (2007). Characterization of natural hybrids of Saccharomyces cerevisiae and Saccharomyces bayanus var. uvarum. FEMS Yeast Res..

[67] Masneuf I, Hansen J, Groth C, Piskur J, Dubourdieu D (1998). New Hybrids between Saccharomyces Sensu Stricto Yeast Species Found among Wine and Cider Production Strains. Appl. Environ. Microbiol..

[68] Naumov GI (2000). Natural Polyploidization of Some Cultured Yeast Saccharomyces Sensu Stricto: Auto- and Allotetraploidy. Syst. Appl. Microbiol..

[69] Sipiczki M (2008). Interspecies hybridization and recombination in Saccharomyces wine yeasts. FEMS Yeast Res..

[70] Stebbins GL (1940). The Significance of Polyploidy in Plant Evolution. Am. Nat..

[71] Babcock, E. B. (Ernest B., 1877, B., Stebbins, G. L. (George L. & 1906-. American species of Crepis. (1938).

[72] Sanna V, Pretti L (2015). Effect of wine barrel ageing or sapa addition on total polyphenol content and antioxidant activities of some Italian craft beers. Int. J. Food Sci. Technol..

[73] Mortimer RK (2000). Evolution and Variation of the Yeast (Saccharomyces) Genome. Genome Res..

[74] Fundyga RE, Lott TJ, Arnold J (2002). Population structure of Candida albicans, a member of the human flora, as determined by microsatellite loci. Infect. Genet. Evol..

[75] Almeida P (2015). A population genomics insight into the Mediterranean origins of wine yeast domestication. Mol. Ecol..

[76] Wang Q-M, Liu W-Q, Liti G, Wang S-A, Bai F-Y (2012). Surprisingly diverged populations of Saccharomyces cerevisiae in natural environments remote from human activity. Mol. Ecol..

[77] Sniegowski PD, Dombrowski PG, Fingerman E (2002). Saccharomyces cerevisiae and Saccharomyces paradoxus coexist in a natural woodland site in North America and display different levels of reproductive isolation from European conspecifics. FEMS Yeast Res..

[78] Sampaio JP, Gonçalves P (2008). Natural Populations of Saccharomyces kudriavzevii in Portugal Are Associated with Oak Bark and Are Sympatric with S. cerevisiae and S. paradoxus. Appl. Environ. Microbiol..

[79] Corte, L. *et al*. Effect of pH on potassium metabisulphite biocidic activity against yeast and human cell cultures - ScienceDirect. (2012). Available at: https://www.sciencedirect.com/science/article/pii/S0308814612004852. (Accessed: 31st January 2018). 10.1016/j.foodchem.2012.03.025 25005950

[80] Divol B, Du T, Duckitt E (2012). Surviving in the presence of sulphur dioxide: Strategies developed by wine yeasts. Appl. Microbiol. Biotechnol..

[81] Ibeas JI, Lozano I, Perdigones F, Jimenez J (1996). Detection of Dekkera-Brettanomyces strains in sherry by a nested PCR method. Appl. Environ. Microbiol..

[82] Schuelke M (2000). An economic method for the fluorescent labeling of PCR fragments. Nat. Biotechnol..

[83] Kamvar ZN, Tabima JF, Grünwald NJ (2014). Poppr: an R package for genetic analysis of populations with clonal, partially clonal, and/or sexual reproduction. PeerJ.

[84] Bruvo R, Michiels NK, D’souza TG, Schulenburg H (2004). A simple method for the calculation of microsatellite genotype distances irrespective of ploidy level. Mol. Ecol..

[85] Paradis E, Claude J, Strimmer K (2004). APE: Analyses of Phylogenetics and Evolution in R language. Bioinformatics.

[86] Jombart T (2008). adegenet: a R package for the multivariate analysis of genetic markers. Bioinformatics.

[87] Frichot E, François O (2015). LEA: An R package for landscape and ecological association studies. Methods Ecol. Evol..

[88] Höhna S, May MR, Moore BR (2016). TESS: an R package for efficiently simulating phylogenetic trees and performing Bayesian inference of lineage diversification rates. Bioinformatics.

[89] Henderson L (1908). Concerning the relationship between the strength of acids and their capacity to preserve neutrality. Am. J. Physiol.-Leg. Content.

