# Supplementary material for: Brettanomyces bruxellensis population survey reveals a diploid-triploid complex structured according to substrate of isolation and geographical distribution
Source: Sci Rep. 2018 Mar 7;8:4136. doi: 10.1038/s41598-018-22580-7 (PMC5841430; doi:10.1038/s41598-018-22580-7)
Supplement: Supplementary file 1 — Supplementary information [file 41598_2018_22580_MOESM1_ESM.pdf]

Brettanomyces bruxellensis population survey reveals a diploid-triploid complex structured according to substrate of isolation and geographical distribution

Marta Avramova[1,8,*], Alice Cibrario[1], Emilien Peltier[1], Monika Coton[2], Emmanuel Coton[2], Joseph Schacherer[3], Giuseppe Spano[4], Vittorio Capozzi[4], Giuseppe Blaiotta[5], Franck Salin[6], Marguerite Dols-Lafargue[1,7], Paul Grbin[8], Chris Curtin[9], Warren Albertin[1,10], Isabelle Masneuf-Pomarede[1,11]

[1]Univ. Bordeaux, ISVV, Unité de recherche Œnologie EA 4577, USC 1366 INRA, Bordeaux INP, 33140 Villenave d'Ornon, France

[2]Université de Brest, EA 3882, Laboratoire Universitaire de Biodiversité et Ecologie Microbienne, ESIAB, Technopôle Brest-Iroise, 29280 Plouzané, France

[3]Université de Strasbourg, Centre National de la Recherche Scientifique, Génétique Moléculaire, Génomique, Microbiologie, Unité Mixte de Recherche, 7156, Strasbourg, France

[4]Department of the Science of Agriculture, Food and Environment, University of Foggia, Foggia, Italy

[5]Department of Agricultural Sciences, Division of Vine and Wine Sciences, University of Naples Federico II, Viale Italia - 83100 Avellino (Italy)

[6]INRA, UMR Biodiversité Gènes et Ecosystèmes, PlateForme Génomique, 33610 Cestas, France

[7]Bordeaux INP ISVV EA 4577, F-33140 Villenave d'Ornon, France

[8]School of Agriculture, Food and Wine, The University of Adelaide, PMB 1, Glen Osmond, SA 5064, Australia

[9]Department of Food Science and Technology, Oregon State University, 100 Wiegand Hall, Corvallis, Oregon 97331-6602, USA

[10]ENSCBP, Bordeaux INP, 33600 Pessac, France

[11]Bordeaux Sciences Agro, 33170 Gradignan, France

*avramova.marta@gmail.com

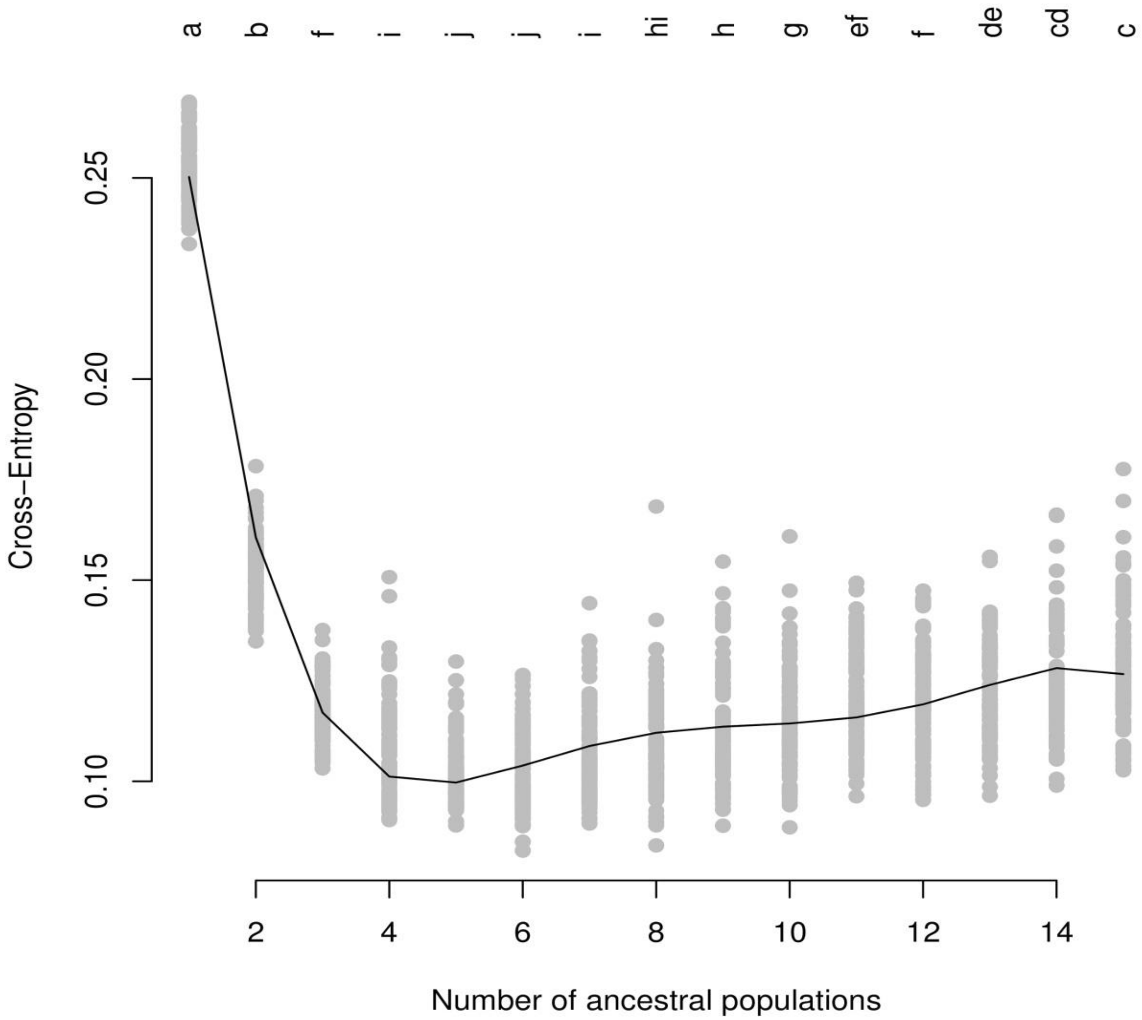

Supplementary Figure S1. Entropy analysis for estimating the number of ancestral populations that explains the genotypic data in the best way. Different letters correspond to the significance groups according to Kruskal-Wallis (α=5%).

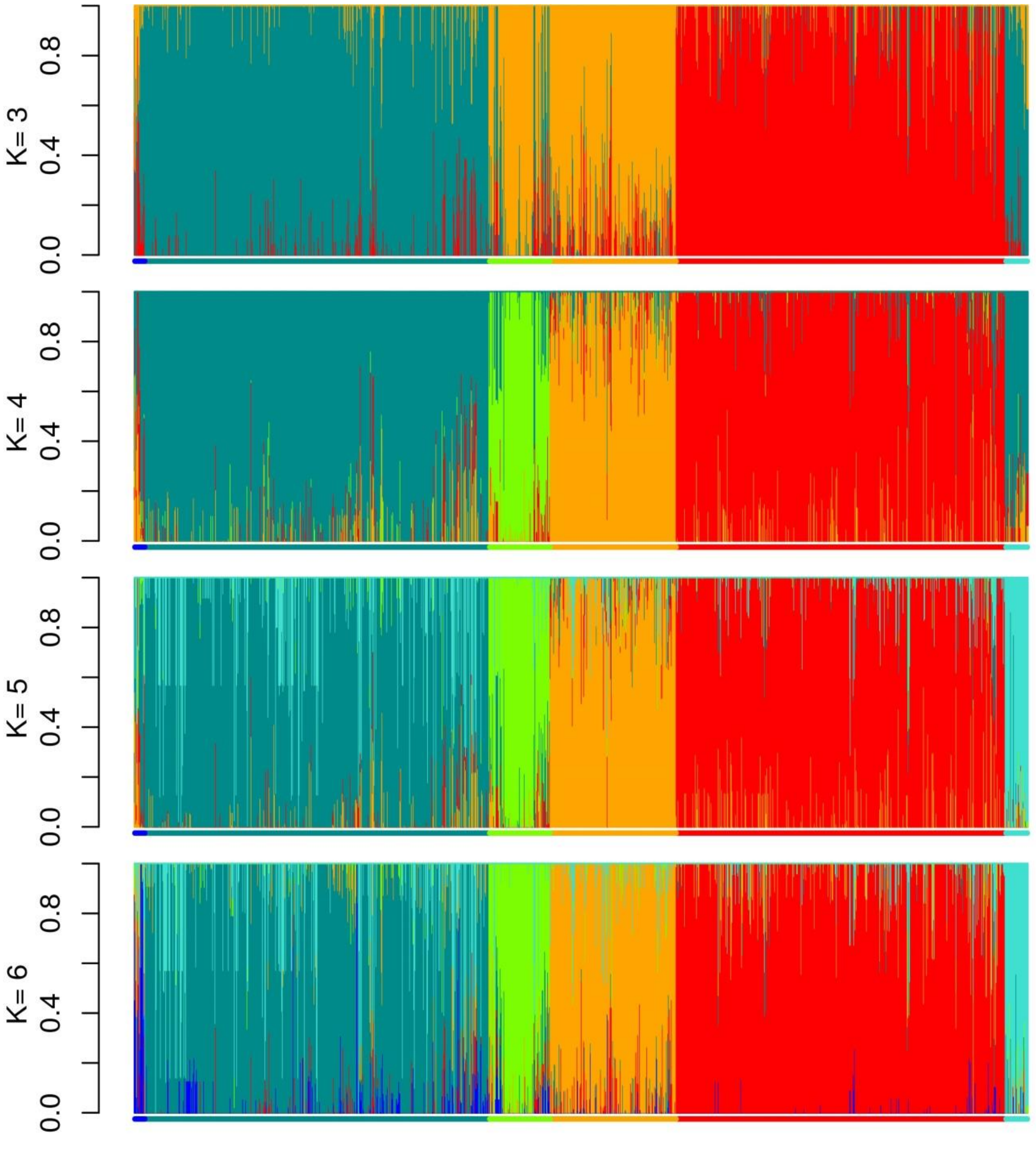

Supplementary Figure S2. STRUCTURE plots for K= 3, 4, 5, and 6. Each bar represents a strain and the colour of the bar represents the estimated ancestry proportion of each of the K clusters. The same colour code is kept as in Figure 1-3. K=5 is the number of ancestral population with lowest entropy (see Supplementary Figure S4 online).
